# Sex classification using exocranial surfaces in a multi-population sample

**DOI:** 10.1007/s00414-025-03694-w

**Published:** 2025-12-23

**Authors:** Markéta Hamanová Čechová, Barbora Suchá, Ján Dupej, Jaroslav Brůžek, Chiara Villa, Radoslav Beňuš, MennattAllah Hassan Attia, Šárka Bejdová, Ahmed Habiba, Tereza Meinerová, Jana Velemínská

**Affiliations:** 1 Department of Anthropology and Human Genetics, Faculty of Science, Charles University, Viničná 7, Prague 2, 128 44 Czech Republic; 2 Section of Forensic Pathology, Department of Forensic Medicine, University of Copenhagen, Frederiksvej V’s Vej 11, Copenhagen, 2100 Denmark; 3 Department of Anthropology, Faculty of Natural Sciences, Comenius University in Bratislava, Ilkovičova 6, Bratislava 4, 84215 Slovakia; 4 Department of Forensic Medicine and Clinical Toxicology, Faculty of Medicine, Alexandria University, Champollion street, Al Mesallah Sharq, Qesm Al Attarin, Alexandria, 21517 Egypt; 5 Department of Radiology, Faculty of Medicine, Alexandria University, Champollion street, Al Mesallah Sharq, Qesm Al Attarin, Alexandria, 21517 Egypt

**Keywords:** Forensic anthropology, Skull, Population specificity, Sex estimation, Sexual dimorphism, 3D imaging methods

## Abstract

The accurate individual identification of skeletal remains is indispensable in forensic contexts. The skull serves as an important source of information about the sex of human skeletal remains, and many different approaches have been published. High method success and reliability are prerequisites for the legal utilisation of results. However, the population specificity of variable sexual dimorphism typically reduce effectiveness. This study presents a verification of an innovative classification model using the exocranial surface across a multi-population sample. This sex estimation method proved to be highly reliable and accurate for Central European populations, achieving high accuracy rates for Czech (96%) and Slovak (92%) samples. The French sample had an accuracy of 90%, demonstrating the method’s effectiveness in Southern European contexts. Prediction using the combined data from these three populations achieved a cross-validation accuracy of 91.74%. When this classifier model was applied to Egyptian crania, the accuracy dropped to 82%, and when applied to crania from a Danish dataset to 80%. The reasons for the failure of the classifier are the smaller degree of sexual dimorphism among Danes, and the more distinct morphological differences in males and females among Egyptians. These lower accuracy rates indicate that the classifier’s reliability diminishes when applied to more diverse and geographically distant populations. The classifier does not work well when applied to a population other than that for which it was developed. The method is robust, and requires further refinement to achieve similar reliability across a broader range of populations.

## Introduction

Introduction The sex estimation of unknown skeletal remains is an important part of anthropological examination. Together with assessment of population affinity, skeletal age and stature, it plays key role in the process of personal identification in forensic and bioarchaeological contexts [1–3]. There are three possible approaches for sex estimation: osteology, genomics and proteomics. The first two are traditional, while proteomics is a relatively new method that has been gaining increasing attention from scientists e.g. [4, 5]. Biological anthropology has undergone a significant transformation: since the inception of virtual methods, there has been an increase in innovative strategies for sex identification, including geometric morphometric methods, and the application of machine and deep learning [6–9]. Advances in modern imaging and analytical techniques, including CT, MRI and geometric morphometrics have contributed to the enhancement of research capabilities, enabling more precise and comprehensive analyses in various scientific domains [10]. Virtual methods may reduce subjectivity when consistent automated processes are used for data collection and analysis e.g. [11]. This standardisation minimises human error and variability, leading to more reliable and reproducible results [12]. By applying geometric morphometric techniques and virtual methodologies form and shape can be analysed with high precision [13, 14].

The success of sex estimation methods is linked to the extent and manifestation of sexual differences in the skeletal structure [15–17]. The pelvis exhibits relatively high reliability for sex estimation [18–20], but other skeletal regions must be used when the pelvis is damaged or lost. Compared to the pelvis, the skull has a lower level of sexual dimorphism expression that is, moreover, population specific [21, 22], while the skull has been reported to be relatively well-preserved even after centuries. Numerous studies have used the skull for sex estimation with varying levels of accuracy; to achieve a reliable sex estimation from the skull, successful classifications must exceed 80% or 85% accuracy [17, 23–26]. In forensic settings, a minimum threshold of 95% representing the optimal level of accuracy and reliability of biological sex estimation is acceptable, which may vary depending on the condition of the remains available for examination [27–29]. Assessing the balance between the number of classified individuals and the accuracy, some studies consider moving/decreasing the posterior probability value for correct classification to a range from 0.75 to 0.8 e. g. [26, 30–32].

Sexual dimorphism of the human skull results from a combination of genetic factors and environmental influences, typically manifesting around puberty [33, 34]. The evolutionary factors shaping skeletal sexual dimorphism in modern humans, and the geographic and chronological variability of phenotypic differences between sexes, are central research topics in anthropology [35].

An important factor that greatly affects the sexual dimorphism of the skull is population specificity [21, 36–38]: the manifestation of sexual dimorphism can be dissimilar in the same part of the skull for different geographical areas. The population specificity of methods using the skull or part of the skull has been confirmed by several articles [38–44].

The issue of the population specificity of methods can be approached in two ways. The first involves designing methods that are population-specific, but their validity is influenced by the secular trend [45–47], and in many populations there is no suitable set of individuals of known sex on which to develop such a design. The second approach, called global standardization, involves methods designed for a multi-population sample of individuals of known sex, which covers as much variability within the human species as possible [22, 48]. This fact points to the need to test all methods before applying them to another population and verifying their robustness. The subjects of this study were therefore (1) the variability and sexual dimorphism of the exocranial surface of individuals from five different regions of Europe and North Africa; (2) the verification of the sex classification model on population samples not included in the classifier.

## Materials

The material comprises five datasets (Table 1) that contain individuals from different European regions – namely Central Europe (Czech Republic, Slovak Republic), Northern Europe (Denmark) and Southern Europe (France) – and North Africa (Egypt). CT scans of skulls were collected from a total of 618 individuals.

**Table 1:** Populations used in the study and demographic details

Population sample	Number of individuals	Number of females	Number of males
Czech	143	59	84
Slovak	92	55	37
French	103	51	52
Danish	179	89	90
Egyptian	101	50	51
Total	618	304	314

The first examined dataset (CZE) consists of 143 CT images of a Central European population from the Czech Republic. These data were collected and anonymised with the approval of the Ethics Committee at the Radiology Department at Na Homolce Hospital in Prague. Scans of patients were acquired using a Siemens Somatom Sensation 16 scanner.

The second examined dataset (SVK) consists of 92 CT images of a Central European population from Slovakia. This data was collected and anonymised with the approval of the Charles University Ethics Committee. Scans of patients were acquired using a Siemens Somatom Volume Zoom.

The third investigated dataset (FRA), from the original study of Musilová et al. (2016), consists of 103 CT images of a Mediterranean Southern European population from France. These data were collected and anonymised with the approval of the Ethics Committee of the University of Aix-Marseille: Faculty of Medicine in Marseille, France. Scans of patients were acquired using a Siemens Sensation 64 scanner at the Department of Radiology at the North Hospital in Marseille, France.

The fourth studied dataset (DEN) consists of 179 CT images of a Northern European population from Denmark. The CT scanning of autopsied individuals was conducted at the Department of Forensic Medicine, University of Copenhagen, using a Siemens Somatom Definition 64 slice scanner, as part of requisitioned work. The data were anonymized, and 3D models were created using Mimics software and exported in STL format. No ethical permission is necessary when working with imaging.

The fifth investigated dataset (EGY) consists of 101 CT images of a North African population from Egypt. This data was collected and anonymised with the approval of the Ethics Committee at the Alexandria Faculty of Medicine. Scans of patients were acquired using an Aquilion 64, Toshiba Medical Systems.

The data were anonymous, tagged only with the age and sex of the individuals. All CT scans were captured for medical reasons that had not influenced the morphology of the skulls. The skulls were without pathologies or deformities.

## Methods

### Image processing

CT scans were converted into surfaces using Amira and/or Avizo (Thermo Fisher Sciences Inc., Waltham, Massachusetts) and Materialise Mimics (Materialise NV, Leuven, Belgium). Triangle meshes were trimmed in Rapidform XOS (INUS Technology, Inc., Seoul, South Korea) and/or MeshLab (ISTI - CNR, Pisa, Italy). In this step, the process involved manually eliminating the spine and unrelated objects from the dataset. Additionally, the jaw and teeth were excluded due to their inherent variability, and any shadow artifacts arising from dental fillings were also removed. To optimise the computational efficiency and reduce complexity, the surfaces were then simplified to approximately 50,000 triangles.

Exocranial surfaces were acquired in Morphome3cs (Charles University, Prague, Czech Republic). An automated procedure was implemented to minimise user interaction; this involved constructing an axis-aligned cube around the skull, with a side length set to 100 times the distance between the two most distant points of the skull. The cube’s centre and the vertex centroid of the skull were aligned. The set Q consisted of 26 points, including the 8 cube vertices, 12 edge mid-points, and six face centres. The exterior surface of the skull was then generated by removing triangles not visible from at least two points of Q. A point, such as point a, was considered visible from point b if no triangle intersected the line segment ab. This automated processing, conducted in Morphome3cs, took only seconds per skull.

Eight landmarks (Table 2) were manually placed on the cranial surface by a trained anthropologist in Morphome3cs. These landmarks are only used to align the models to each other before statistical analyses. Statistical analyses do not run on them, thus reducing human influence on the results.

**Table 2:** List of used landmarks with Martin’s handbook definitions [67]

Landmark	Definition
Glabella	The most anteriorly projecting point in the midsagittal plane at the lower margin of the frontal bone, which lies above the nasal root and between the superciliary arches
Inion	The most prominent point of the external occipital protuberance
Opisthion	The point at which the midsagittal plane intersects the posterior margin of the *foramen magnum*
Nasospinale	The point at which the midsagittal plane intersects the lowest point of the lower margin of the *apertura piriformis*
Mastoidale dx, sin	The lowest point of the mastoid process (*processus mastoideus*)
Zygomaticofrontale dx, sin	The point at which the inner orbital margin intersects the *sutura zygomaticofrontalis*

Before statistical processing, it was imperative to ensure vertex homology between the surfaces. From the various applicable procedures, the chosen method was coherent point drift-dense correspondence analysis (CPD-DCA) [49]. This approach involves an initial rigid alignment of the meshes using generalised Procrustes analysis (GPA) on landmarks. Subsequently, an automatic non-rigid registration algorithm, coherent point drift (CPD), is applied to deform rigidly aligned surfaces to the template surface, also referred to as the base mesh. Following registration, a closest-point search is employed to identify corresponding vertices, termed quasi-landmarks. Any vertices that cannot be matched are excluded from further processing to prevent unwanted variability from contaminating the results. To reduce sensitivity to landmark placement errors, the surfaces undergo another round of rigid alignment through GPA, this time involving all quasi-landmarks that were not removed in the previous step. This multi-step process ensures that the surfaces are aligned and comparable for subsequent statistical analyses. All statistical and machine learning procedures were carried out using Morphome3cs. Additionally, the R programming language, specifically the package e1071 [50], was used for statistical computing throughout the analysis.

### The evaluation of variability and sex differences

Given the substantial dimensionality of the quasi-landmark coordinate matrix of approximately 100k columns, a high-dimensional principal component analysis [51] was conducted on these coordinates. The aim of this analysis was to reduce the dimensionality while retaining most of the variability present in the dataset. The principal components represent quantities that describe the overall variability of the observed features; the first principal component explains the largest percentage of variability, each subsequent one capturing a smaller part of the variability that was not described by the previous components [52]. The PCA was used to analyse the variability between populations.

Differences in sexual dimorphism were visualised in each population sample. Color maps were constructed by color-coding the distances of the vertices from the mean male and female surfaces after projection to the local surface normal. This projection effectively transforms the vertex distance to the local surface distance.

Removing centroid size provides a way to standardise shape data by removing the effect of size, allowing us to focus on shape when comparing differences between populations. Centroid size was calculated as the square root of the sum of squared distances between each landmark point and the centroid of the configuration. The centroid is essentially the geometric centre of the shape. Therefore, centroid size is a measure of the extent of the shape from its centroid.

Results were quantified by ANOVA (Analysis of Variance) and the Tukey HSD (Honestly Significant Difference) for significance evaluation and p-value determination.

### Sex classification

In the final step of the study, sex classification was performed. Support vector machines (SVM), a robust supervised machine learning approach [53], were employed. The goal was to train a classifier using the principal component scores and the known sex of the skulls. The SVM was trained with a radial kernel, and the training process was repeated for both form and shape scores, considering different numbers of principal components (ranging from 1 to 30). To evaluate the performance of the classifier and identify potential overfitting, leave-one-out cross-validation was employed. This process assessed how well the classifier generalised the information by leaving out one observation at a time for testing, while training on the remaining data. For both form and shape, the analysis involved selecting the lowest number of principal components that produced the highest cross-validation success rate.

Confusion matrices serve as a valuable tool in machine learning and statistical analysis for assessing the performance of classification models. They are especially beneficial in binary classification scenarios, where the outcome can be categorised into two distinct classes, such as true or false, positive, or negative, and so on. By comparing the predicted classifications with the actual outcomes, confusion matrices provide a comprehensive overview of the model’s performance, including measures like accuracy or precision [54]. This detailed evaluation aids in understanding the strengths and weaknesses of the classification model and guides potential improvements.

To calculate the bias, we first need to understand what it represents in the context of a confusion matrix. In the context of classification, bias refers to the tendency of a model to systematically predict one class over the other. Bias can be calculated using the formula (where FP means false positive, and TN means true negative):

$$\mathrm{Bias}=\frac{FP}{FP+TN}$$

## Results

### The evaluation of variability and sex differences

**Fig. 1:**
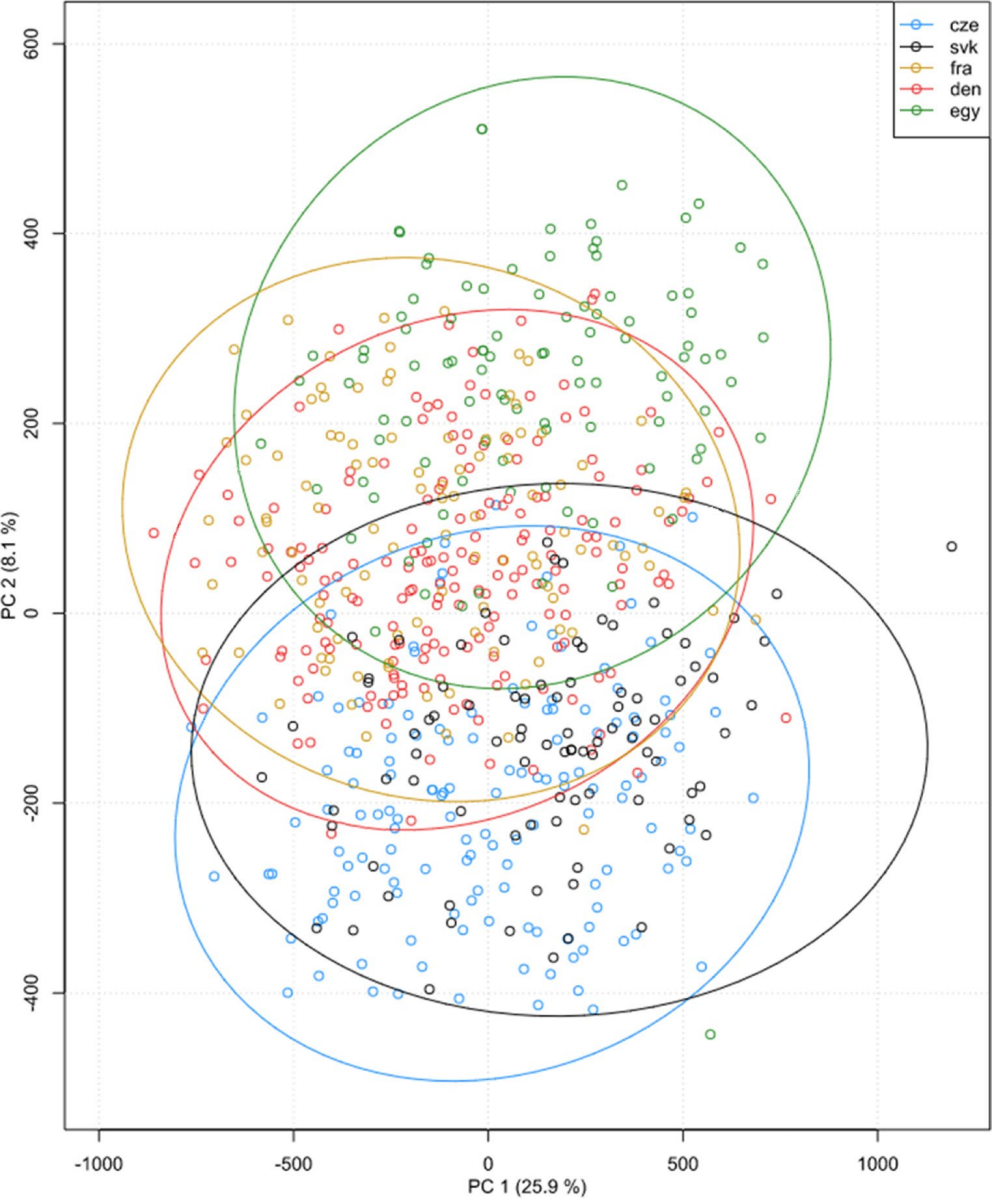
PCA scatter plot depicting all studied datasets. Distribution of the sample in the first two principal components in form. The colours and shapes of the points correspond to different groups (populations) as indicated by the legend: blue circles “cze” (Czech sample); black circles “svk” (Slovak sample); yellow circles “fra” (French sample); red circles “den” (Danish sample); green circles “egy” (Egyptian sample)

A principal component analysis biplot (Fig. 1) was used to visualise the variation in the multivariate dataset. PC1 and PC2 explain 25.9% and 8.1% of the variance in the data respectively, while each point represents an individual specimen. PC1 mainly explains the variability related to the size of the crania; PC2 is responsible for shape changes. There is a significant overlap between the groups, indicating that while there are some differences, the groups are not completely distinct in terms of the first two principal components analysed. The Czech Republic (blue) and Slovakia (black) have considerable overlap, suggesting these populations have similar characteristics in the dimensions captured by PC1 and PC2. Denmark (red) and France (yellow) are somewhat intermixed and are moved towards positive PC2 values compared to the Czech and Slovak samples. Egypt (green) shows clustering towards the positive end of PC2. A few outliers can be noted, such as some Egyptian specimens (green) that are far from the main cluster, indicating the unique characteristics in those samples.

**Fig. 2:**
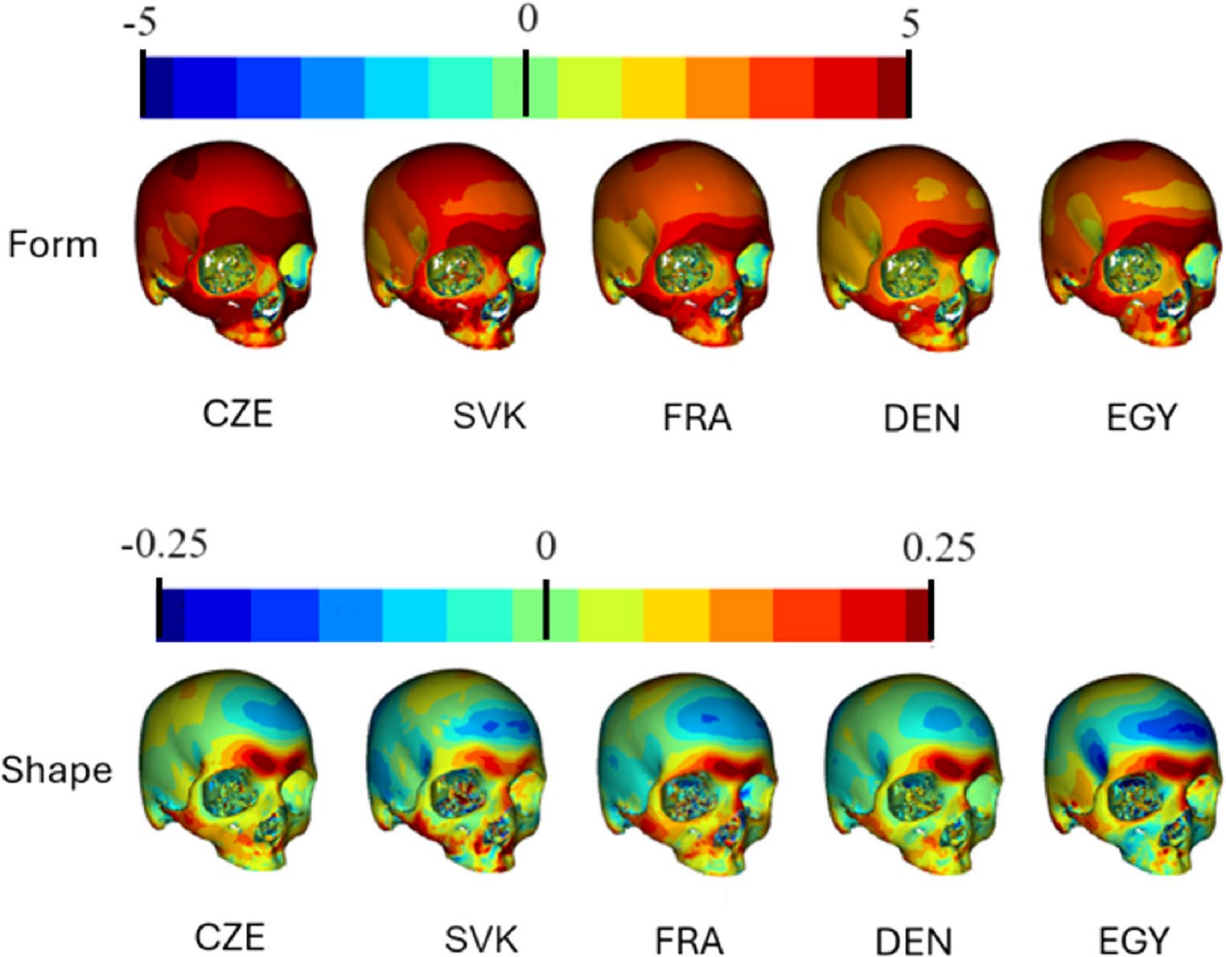
Comparative visualisations of anterior and lateral part of craniums from different regions, showcasing both form (in millimetres) and shape (relative) differences. Red represents the most prominent areas in males in comparison to females. *Abbreviations*: CZE Czech sample, SVK Slovak sample, FRA French sample, DEN Danish sample, EGY Egyptian sample

**Fig. 3:**
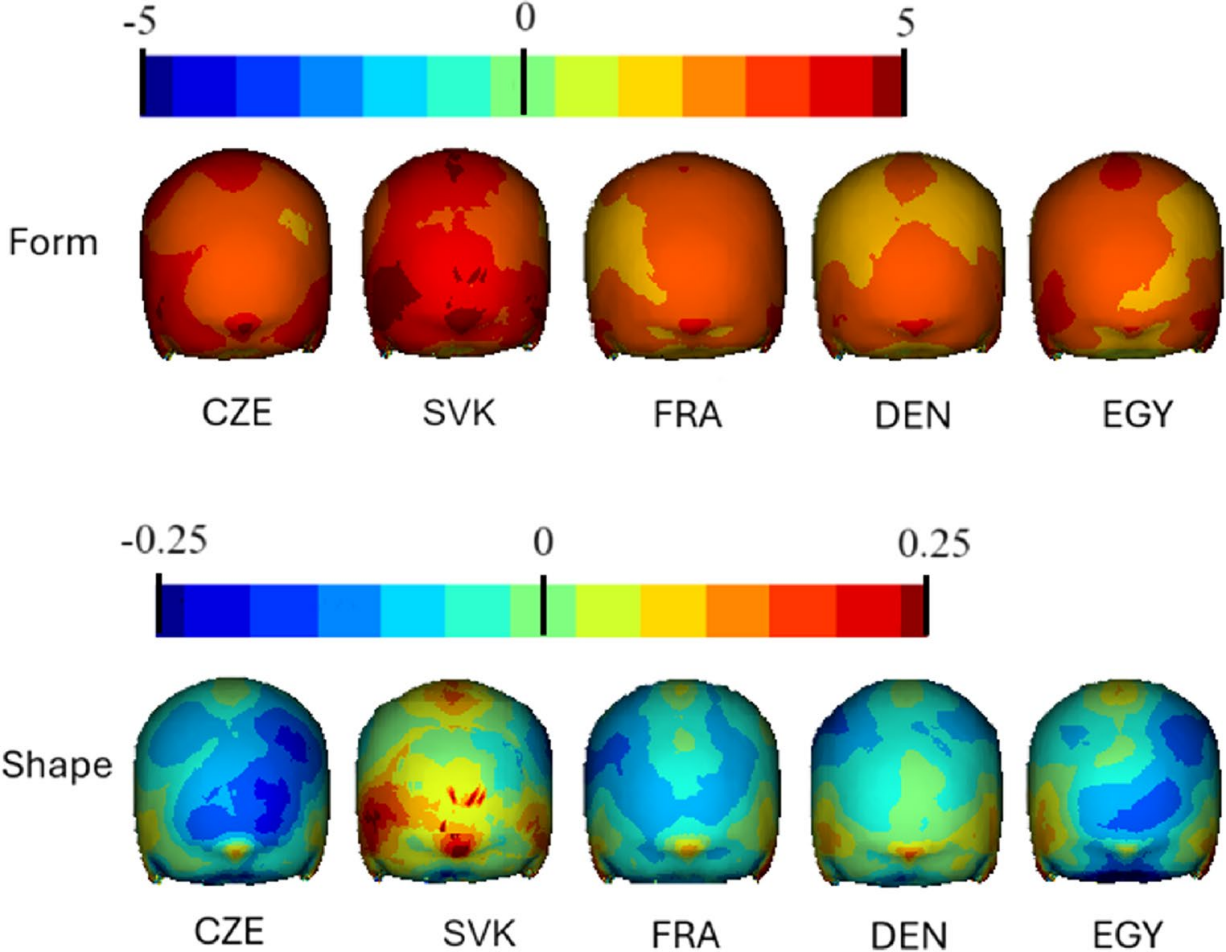
Comparative visualisations of posterior part of craniums from different regions, showcasing both form (in millimetres) and shape (relative) differences. Red represents the most prominent areas in males in comparison to females. *Abbreviations*: CZE Czech sample, SVK Slovak sample, FRA French sample, DEN Danish sample, EGY Egyptian sample

The comparative visualisations of sex differences of crania from different regions are shown in Figs. 2 and 3, showcasing both form and shape differences. The regions represented are the Czech Republic, Slovakia, France, Denmark and Egypt. Each row of crania represents a different aspect of comparison: form (top row) and shape (bottom row). Red represents areas which were more prominent in males, while more prominent morphological features in females are depicted in blue.

Figure 2 shows the anterior and lateral views. In the form analysis, the most pronounced sexual differences are evident in the Czech sample. In the area of neurocrania, males are very prominent especially in the forehead, but also in the lateral regions. In the facial area, the greatest differences are seen in the maxilla and zygomatic bone. Slovaks show the greatest similarity in sexual dimorphism with the Czech sample, but with a more moderate expression of sexual differences. The glabellar region consistently shows dark red areas across all regions in terms of form. The differences in shape are quite similar in all five populations with male prominences in the glabellar region or the lateral parts of face, and female prominences in the frontal tubers. Egyptian crania are distinguished from other populations in shape by the protrusion of the maxillary region in females.

Figure 3 shows the posterior part of the cranium. In the form analysis, the most pronounced sexual differences are evident in the Slovak sample, in which males are very prominent in comparison with females. Czechs are probably the most similar to the Slovak sample, but the sex differences are more moderate. In other samples, the manifestation of sexual dimorphism is slightly milder still, but the posterior part of the cranium is more pronounced in males than in females. In the shape analysis, the Slovaks are very different from the other populations studied. Slovak skulls are dominated by areas in which males are prominent; conversely, the other studied populations show a predominance of females. The prominence of the female skull is most pronounced among the Czechs and Egyptians, followed by the French. In the Danish group, the prominence of females is still evident, but only with a milder manifestation, and in the medial part of the occipital bone, males and females overlap. In all skulls, however, there is a more pronounced protuberantia occipitalis externa in males.

Results were quantified by ANOVA, which is used to determine if there are significant differences between groups (Table 3). The p-value for sex and sample are extremely low (*p* < 0.001), indicating that the effect of sex and population on the dependent variable are highly statistically significant. There are statistically significant differences in the dependent variable between males and females as well as across different populations. However, the interaction of sex and population is not significant. The result of this test may indicate that there is no significant difference in sexual dimorphism between populations.

**Table 3:** The *p*-values for the main effects of sex, sample, and their interaction from the ANOVA test

Source	*p*-value
Sex	0.000*
Sample	0.000*
Sex: sample	0.157

* *p* < 0.001 (highly significant result)

The Tukey HSD test results for male cranial samples from different populations are interpreted in Table 4. Significant differences in male cranial dimensions are observed mainly between Egyptian and other populations, indicating distinct cranial features. Significant differences in female cranial dimensions are interpreted in Table 5. Highly statistically significant differences were also observed mainly between Egyptian and other populations. The other pairwise comparisons mostly show no significant differences, except for the Slovakian sample, which displays notable differences compared to the other groups with the exception of the Czech sample.

**Table 4:** The Tukey HSD test results for males: *p*-values for each pairwise comparison of male cranial samples from different populations

	CZE	SVK	FRA	DEN	EGY
CZE	-	0.106	1.000	0.356	0.000***
SVK	0.106	-	0.126	0.833	0.003**
FRA	1.000	0.126	-	0.403	0.000***
DEN	0.356	0.833	0.403	-	0.000***
EGY	0.000***	0.003**	0.000***	0.000***	-

Level of statistical significance: *** *p* < 0.0010; ** *p* < 0.01; * *p* < 0.05

**Table 5:** The Tukey HSD test results for females: *p*-values for each pairwise comparison of female cranial samples from different populations

	CZE	SVK	FRA	DEN	EGY
CZE	-	0.638	0.219	0.438	0.000***
SVK	0.638	-	0.007**	0.014*	0.018*
FRA	0.219	0.007**	-	0.962	0.000***
DEN	0.438	0.014*	0.962	-	0.000***
EGY	0.000***	0.018*	0.000***	0.000***	-

Level of statistical significance: *p* < 0.0010 ‘***’; *p* < 0.01 ‘**’; *p* < 0.05 ‘*’

**Fig. 4:**
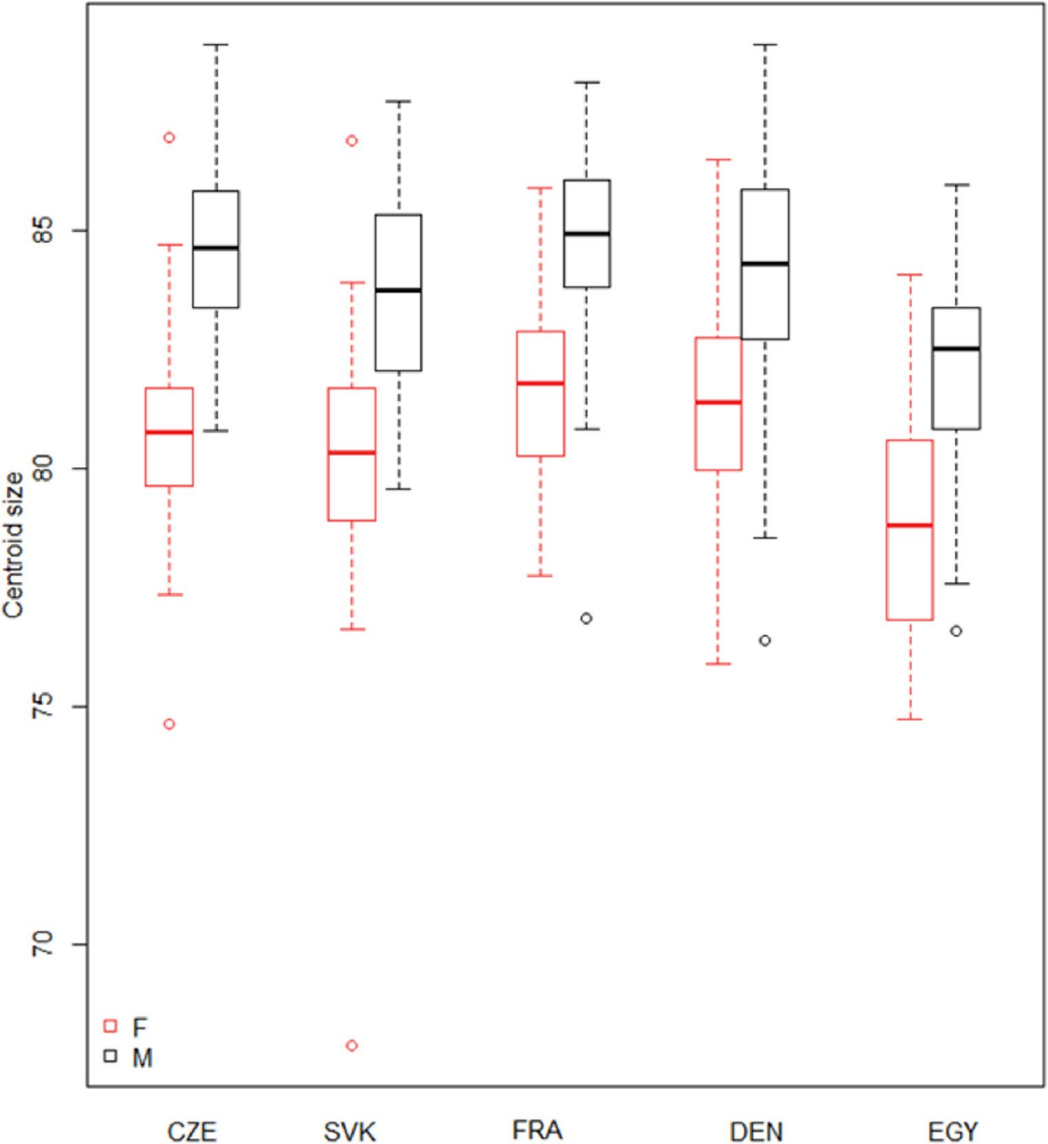
Box plot comparing the centroid sizes of male and female skulls across five different regions (CZE, SVK, FRA, DEN, EGY). *Abbreviations*: CZE Czech sample, SVK Slovak sample, FRA French sample, DEN Danish sample, EGY Egyptian sample

Figure 4 shows the centroid size for each studied population, separately for females and males. The greatest differences between males and females are seen in the Czech sample, while the smallest sex differences are found among the Danes. Even so, the variability within each group is quite similar, indicating a consistent spread of centroid sizes within these populations. The median centroid sizes for the Czech, Slovak, French and Danish samples are quite similar, while slightly lower for the Egyptian sample. The slightly lower median centroid size of the Egyptian compared to the other groups might suggest a smaller overall size for specimens from this population. On the other hand, sexual dimorphism is evident among Egyptians. Outliers are present in the Czech and Slovak populations, indicating that there are a few specimens with significantly smaller centroid sizes in these groups. The plot shows that while there are some differences in centroid sizes among the groups, particularly with the Egyptian having a slightly smaller median size, the overall size distributions are fairly consistent.

### Sex classification

The sex classification results are presented in Table 6. The classifier performed very well on the Czech dataset, with an accuracy of 96.15%, the highest among the individual populations. The accuracy for Slovaks is also high at 92.25%. The classifier accuracy for the French population is 90.29%, indicating good performance.

**Table 6:** Sex classification accuracy

Sample	SVM + CV
CZE	96.15%
SVK	92.25%
FRA	90.29%
CZE + SVK + FRA	91.74%
DEN	80.45% *
EGY	82.18% *

* Sex classification accuracy using the classifier trained on the CZE + SVK + FRA dataset

**Fig. 5:**
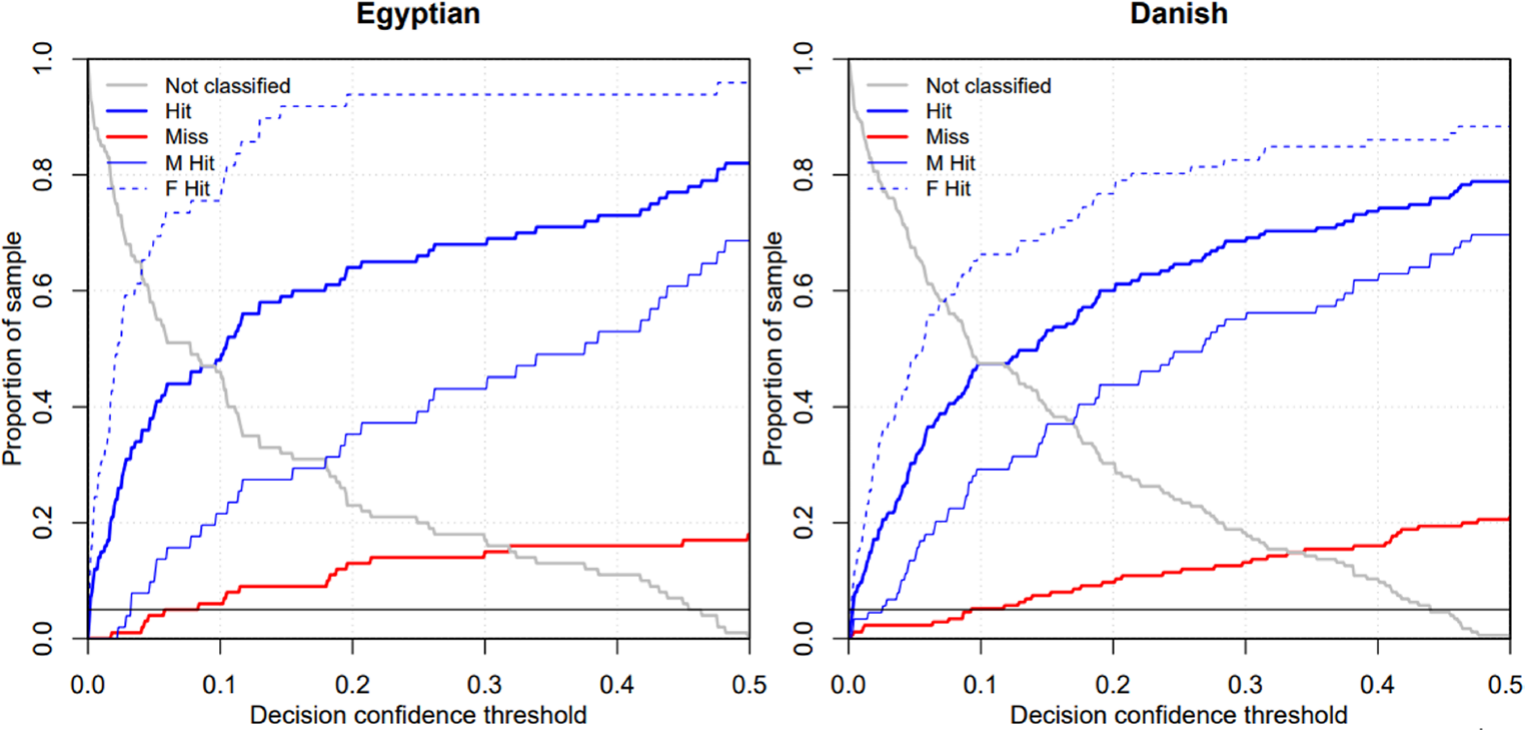
Dependence of classifier success (trained on CZE + SVK + FRA) on the decision confidence threshold. Changing the threshold will cause a change in the number of correctly determined (Hit), incorrectly determined (Miss), and undetermined (Not classified). The thin lines indicate the success rates for males (M Hit) and females (F Hit). The black horizontal line indicates 5%, and when it meets the red line, the model incorrectly determines 5% of the test sample. *Abbreviations*: CZE Czech sample, SVK Slovak sample, FRA French sample

Based on the multi-population dataset of the Czech, Slovak and French data files with similar classification success rates, a classifier model was created. The prediction achieved a cross-validation accuracy of 91.74% on these combined data. Using this model to classify Egyptian crania yielded an accuracy of 82.18%, and to classify crania from the Danish dataset an accuracy of 80.45%. According to posterior probability results with a PP 0.9 threshold (Table 7), the classifier is more successful in correctly classifying Egyptian samples (57.4%) than Danish ones (46.4%). The misclassification rates are relatively low for both populations, with Egyptians at 3.0% and Danes at 3.3%, indicating that when the model does make an error, it is not dramatically high. The classifier performs moderately well, with a higher correct classification rate of Egyptian and Danish crania; however, a substantial number of samples from both populations could not be classified, indicating potential limitations in the model’s ability to handle these datasets. The low misclassification rates are promising, suggesting that the model is relatively reliable when it does make a classification decision. The success of the classifier varies depending on the decision confidence threshold (Fig. 5). When this threshold is shifted, the number of correctly determined, incorrectly determined and undetermined crania changes. For both Egyptian and Danish crania, it is evident that the classification model identifies females more successfully than males (especially among the former).

**Table 7:** Posterior probability results with a high threshold (pp 0.90) (Avent et al. 2021)

	EGY	EGY M	EGY F	DEN	DEN M	DEN F
Correctly classified	57.4%	33.3%	82.0%	46.4%	27.8%	65.2%
Not classified	39.6%	60.8%	18.0%	50.3%	65.6%	34.8%
Incorrectly classified	3.0%	5.9%	0.0%	3.3%	6.6%	0.0%

*Abbreviations*: EGY Egyptian sample, DEN Danish sample, *M* male, *F* female

**Fig. 6:**
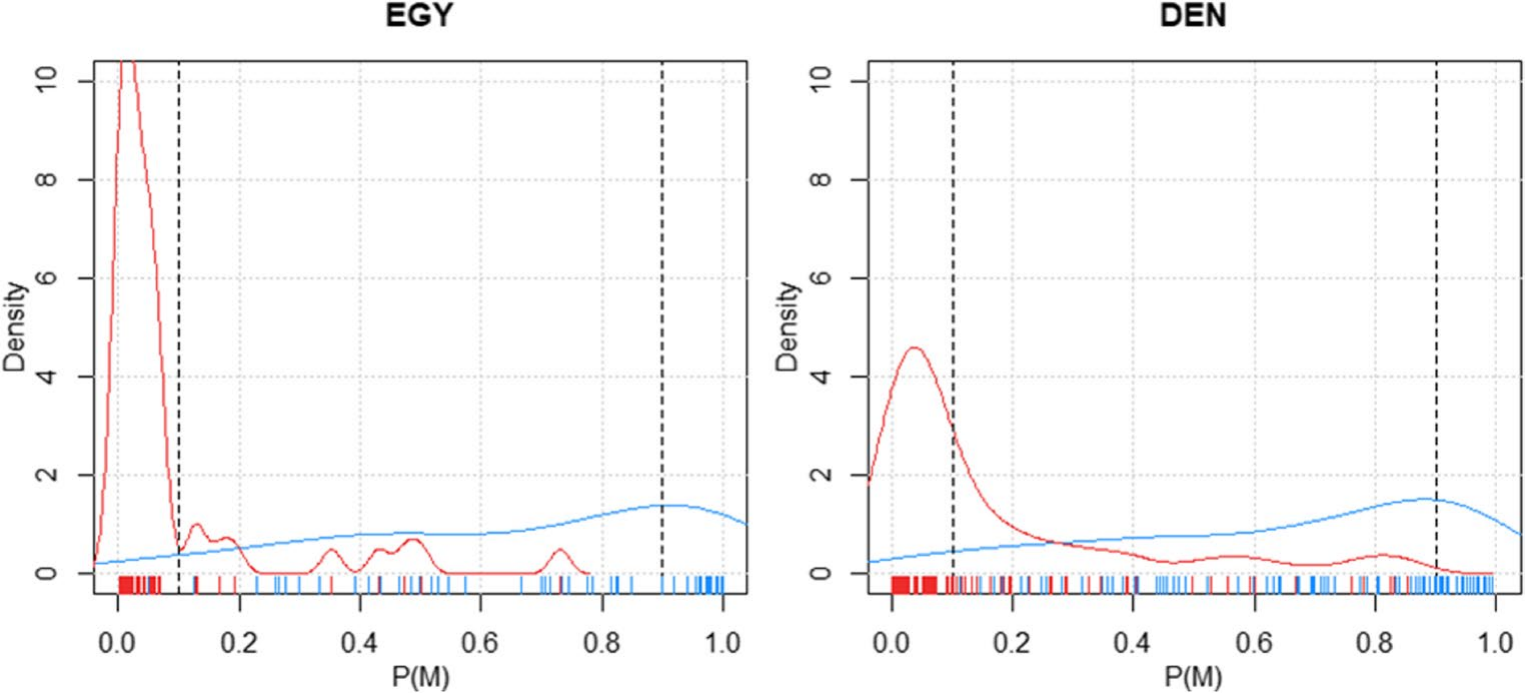
Density based on the estimated probability that an individual is male in Egypt and Denmark sample. Classifier trained on CZE + SVK + FRA. Red density curve shows the distribution of probabilities for females and blue density curve shows the distribution of probabilities for males. The small ticks at the bottom (rug plots) indicate individual data points. Each tick represents a specimen’s probability of being classified as male. *Abbreviations*: CZE Czech sample, SVK Slovak sample, FRA French sample, DEN Danish sample, EGY Egyptian sample

Density plots show the probability distributions of a variable for two populations: Egyptians (EGY) and Danes (DEN). Figure 6 depicts the estimated probability that an individual is male. The X-axis represents the probability of being classified as male P(M); values range from 0 to 1, where 0 indicates a very low probability and 1 a very high probability. The Y-axis represents the density of the data points at different probability values; higher peaks indicate more frequent occurrences of certain probabilities within the sample. Probability distributions are indicted by a red density curve for females and a blue one for males. The small ticks at the bottom (rug plots) indicate individual data points; each tick represents a specimen’s probability of being classified as male.

In the Egyptian dataset the density of the red curve is highest at the lower end of the probability scale (around 0.0), indicating that most females have a low probability of being classified as male. The density of the blue curve is higher across a range of probabilities but peaks around 0.1 and 0.5, indicating that some males are being classified with moderate probability, but some have higher probabilities. There is significant overlap between the red and blue curves, suggesting some ambiguity in classification. The model has difficulty distinguishing between males and females in the Egyptian population.

In the Danish dataset the red curve peaks at the lower end (around 0.1), showing that most females are classified with a low probability of being male, which is similar to the Egyptian plot. The blue curve peaks around 0.0 to 0.1, with a small secondary peak at higher probabilities, indicating a slightly more distinct separation than in the Egyptian population. There is also overlap here, but it is less pronounced than in the Egyptian plot. This suggests that the classifier performs slightly better for the Danish population, but still has some misclassifications. These density plots suggest that while the classifier can generally distinguish between sexes, there are significant instances of overlap where the classifier is less certain, especially in the Egyptian population.

## Discussion

This study focused on the sex estimation of individuals using the external morphology of the human cranium in a global sample by applying advanced methods of geometric morphometrics. Sex estimation using the exocranial surface methodologically builds on the research of Musilová et al. [55] and presents a verification of this innovative classification model across a multi-population sample.

The sex differences in the Czech, Slovak, French, Danish and Egyptian samples in form and shape are shown in the colour map (Figs. 2 and 3). This presents all five datasets’ generally larger significance in form, indicating size differences in specific regions of the cranium. Shape analysis showed a more differentiated range of significance, revealing areas that are more about the geometric configuration than size. These results are consistent with other studies, which advocate for using shape instead of size [13, 56]. These findings were expanded by the results from other analyses, such as PCA and centroid size plots, resulting in a better understanding of how the classifier performs. The Egyptian crania are generally smaller, and the Danish crania appear to exhibit less pronounced sexual dimorphism in size. This variability in cranium size and sexual dimorphism across different populations presents a challenge for the model in making accurate classifications, particularly with the Danish samples. The presence of outliers in some groups suggests individual variability, but removing the outliers did not cause any significant differences in the results.

The results of the sex classification show varying degrees of accuracy in sex estimation across different populations, demonstrating the effectiveness of the developed method in form. The Czech sample achieved the highest accuracy at 96.15%, suggesting that the method is particularly effective for this group. This high level of accuracy indicates that the cranial morphological features used for sex estimation are well-defined and distinct in the Czech population, making it easier to distinguish between sexes. The Slovak sample showed a high accuracy of 92.25%, indicating that the method is robust and reliable for this population as well. This result highlights the method’s consistency and applicability across different Central European populations. The French sample had an accuracy of 90.29%, demonstrating the method’s effectiveness in Southern European contexts. Based on the global Czech, Slovak and French datasets having similar classification success rates, a classifier model was trained. When this combined model was used to classify Egyptian craniums, the accuracy was 82.18%. The Danish sample exhibited the lowest accuracy at 80.45%. The exclusion of Egyptian and Danish datasets from the training process may have contributed to the lower accuracy observed in these populations [57]. In comparison to our findings, previous studies have demonstrated varying degrees of accuracy in sex estimation using different cranial parameters and methodologies. Kimmerle [58] reported an accuracy rate of 87–90% after incorporating centroid size analysis. Similarly, Green & Curnoe [59] achieved an accuracy of 86.8% in sex estimation for Southeast Asian skulls when both shape and centroid size were included in the discriminant analysis. Gillet et al. [60] demonstrated a significant improvement in the accuracy of identifying sexual dimorphism through advanced analysis methods; their study achieved a remarkable accuracy rate of 97.7% for the cranium and 84.2% for the mandible, surpassing the results obtained through traditional metric analysis.

The results show that the suggested classification model is much more successful at classifying females than males in populations that were not included in the training sample. Using a PP 0.9 threshold, 82% of females in the Egyptian sample were correctly classified, compared to 33.3% of males. Similarly, the success rate was 65.2% for females and 27.8% for males in the Danish sample. This suggests that the model is unable to adequately capture or generalise the complex variability observed in males across different populations. This reduces the effectiveness of the classifier when dealing with presumed male human remains, thereby increasing the risk of misclassification. Caution should be exercised when interpreting the classification of presumed male skulls.

In forensic anthropology, the accurate classification of skeletal remains often relies on population-specific data to account for variations in bone structure and size. When working with diverse populations, such as the Egyptian and Danish samples in this study, the model must contend with differing morphological characteristics. Egyptian crania tend to be smaller on average, which can influence the model’s ability to correctly identify sex based on size-related features. Similarly, Danish crania show reduced sexual dimorphism in size, meaning the differences between male and female crania are less pronounced. This reduced dimorphism makes it more challenging for the model to distinguish between sexes accurately. The Tukey HSD test results reveal that significant differences in cranial measurements are predominantly found between the Egyptian samples and other populations, for both males and females. This indicates distinct cranial features in the Egyptian population compared to the others. The findings suggest that while the sex estimation method is highly accurate within certain populations, there are notable variations when applied across different geographical groups. These results emphasise the importance of considering population-specific traits in forensics, and support the ongoing development of more robust and adaptable methods for sex estimation [22]: including data from both Egyptian and Danish populations during the training phase, for instance, could improve the model’s ability to generalise and maintain higher accuracy across diverse groups [57]. Another approach for designing a classifier is to train the classification algorithm based on shape alone. It seems that shape-related characteristics may be overshadowed by size-related ones. The variability in the dataset is primarily explained by PC1, which accounts for a quarter of the total variability and mainly explains the variability related to skull size. This may cause the classifier to adapt to the population-specific size allometry present in the training set rather than identifying shape-related characteristics. This may signify that the predictive potency of independent shape variables remains unquantified, which is problematic given the significance of these variables for sex classification in a forensic context. Further research is needed to determine whether including size in the classifier introduces bias when classifying different populations. The classification algorithm and all related calculations could be developed based solely on shape, and then validated using geographically diverse groups.

Despite the frequent use of cranial characteristics to estimate sex, publications focused on the accuracy of methods and their reliability at the individual level pay little attention to the use and interpretation of posterior probability. A comparison by Avent et al. (2022) showed that below the 0.75 PP threshold, sex estimation was no better than chance, and specimens with PPs below this level should therefore be classified as indeterminate. Those with PPs between 0.75 and 0.84 were significantly better than chance, but should be considered with less confidence than those in the 0.85–1.00 PP range [26]. As highly reliable results are required for forensic studies, we opted for a PP threshold value of 0.9. This classifier is more successful at correctly classifying Egyptian samples (57.4%) than Danish ones (46.4%). The misclassification rates are relatively low for both populations, with Egyptians at 3.0% and Danes at 3.3%. This means that setting a high threshold leads to a low error rate. However, it will also leave a large number of undetermined cases. When the PP threshold is set at 0.9, the proportion of “unclassified” cases reaches 50.3% for the Danish sample and 39.6% for the Egyptian sample. The proportion of cases in which the classifier cannot reliably make a decision is extremely high. However, when it comes to forensic applications, our priorities are reliability and minimising the risk of errors.

Global equations are often inadequate for sex estimation aimed at individual identification, although they can be improved by using large, diverse reference samples to ensure broad representation and reduce bias [61]. While global equations may achieve acceptable accuracy rates, they do not accurately represent the sexual dimorphism of each population. Therefore, for precise individual identification, it is preferable to use population-specific equations when available. Although it is unrealistic to develop population-specific standards for every method and bone, research can prioritize the most reliable skeletal elements [22]. One way to address population variation in sexual dimorphism is to create global databases of trait scores and skeletal measurements, and to ensure that these databases and the resulting equations are accessible to everyone (e.g. FORDISC 3.1, Osteoware, SexEST or MorphoPASSE).

In addition, accurate sex estimation often hinges on consideration of both the size and shape characteristics of skeletal remains. While shape can provide valuable information, such as sexually dimorphic features in facial structure or cranial morphology, the current findings suggest that size may have a more substantial influence on classification outcomes in this particular model. To improve the accuracy and reliability of sex estimation models, it is essential to develop approaches that account for population-specific size variations while also considering shape-related features. This may involve refining existing models to incorporate additional parameters or implementing advanced statistical techniques to better capture the complexities of size distributions across diverse populations [13, 56, 62, 63].

Each sex classifier should be tested on a population for which it was not originally developed before use – but it should also be tested whenever it is applied to a group of individuals that differs in some way from the reference dataset. When applied to a genealogical sample, a classifier may fail because of reduced variability between biologically related individuals due to genetic factors [64]. Also, using a method that has been successful on a recent population may not work as well on historical data [65]. This finding highlights the need for further refinement to increase accuracy in bioarchaeological applications.

The current findings suggest that size may have a very substantial effect on classification outcomes. By prioritizing shape, the method may achieve more accurate and reliable classification outcomes. To further enhance accuracy, future studies could explore the inclusion of additional skeletal features beyond the exocranial surface; incorporating other structures might provide complementary data that enhance the overall reliability of sex estimation. By addressing these research directions, future studies can build on the findings of this thesis to develop even more accurate, reliable, and universally applicable methods for sex estimation from skeletal remains.

A crucial issue is the applicability of the proposed classifier in practice. The use of geometric morphometric methods has been on the rise in recent years. Their advantages are their ability to capture subtle shape differences, analyze shape independent of size, and integrate size and shape together. Beyond sex classification, geometric morphometry also provides an opportunity to more precisely study the differences occurring between males and females [66]. However, the success of the methods on known data does not mean that they will also be successful on unknown cases. Many methods remain only at the theoretical level. It is important to transfer the knowledge gained in research into a practical application in the field that is freely available, so no matter the working environment, one can apply the method. The practical application of our classifier requires a more complex procedure and we cannot currently guarantee its success for practical use on an unknown sample. The results show the limits of our classifier and also set expectations for future practical use.

## Conclusions

This sex estimation method proved to be highly reliable and accurate for Central European populations, achieving high accuracy rates for Czech (96%) and Slovak (92%) samples. The French sample had an accuracy of 90%, demonstrating the method’s effectiveness in Southern European contexts. However, when a classification model was built from these datasets, this classifier did not perform as well for the Egyptian (82%) and Danish (80%) populations. For both Egyptian and Danish crania, the classification model identifies females more successfully than males. The lower accuracy rates indicate that the classifier’s reliability diminishes when applied to more diverse and geographically distant populations. Thus, while the method is robust and reliable within closely related European groups, it requires further refinement to achieve similar reliability across a broader range of populations.

## Data Availability

The datasets generated during and/or analysed during the current study are available from the corresponding author on reasonable request.
